# MicroRNA in atherothromobosis: is it useful as a disease marker?

**DOI:** 10.1186/s12959-016-0112-2

**Published:** 2016-10-04

**Authors:** Satoshi Fujii, Tomonori Sugiura, Yasuaki Dohi, Nobuyuki Ohte

**Affiliations:** 1 Department of Laboratory Medicine, Asahikawa Medical University, Midorigaokahigashi 2-1-1-1, Asahikawa, 078-8510 Hokkaido Japan; 2 Medical Laboratory and Blood Center, Asahikawa Medical University Hospital, Asahikawa, Japan; 3 Department of Cardio-Renal Medicine and Hypertension, Nagoya City University, Nagoya, Japan

**Keywords:** Coronary artery disease, Plasma microRNA, LDL cholesterol, Smoking, Endothelium

## Abstract

Atherosclerosis is one of the major causes of death. Data from animal experiments suggest that atherosclerosis involves an inflammatory process of the vascular wall under hyperlipidemia. Atherothrombosis can become a serious complication of atherosclerosis leading to acute cardiovascular events such as myocardial infarction and stroke. Clinical applications to use this knowledge remain scarce. The plasma levels of vascular endothelium-enriched microRNA (miRNAs) in patients with atherosclerotic vascular disease could serve as a disease marker. In our laboratory vascular endothelium-enriched miRNA (miR-126) level was analyzed using quantitative RT polymerase chain reaction analysis (qRT-PCR) in plasma from patients with suspected coronary artery disease (CAD) according to the chest symptom or findings of electrocardiogram, or middle-aged male smokers. Endothelial function for peripheral small vessels was assessed using End-PAT 2000 and expressed as reactive hyperemia peripheral arterial tonometry (RH-PAT) index. In patients with suspected CAD miR-126 was not significantly changed in CAD patients. However, miR-126 was decreased in CAD patients who also have high levels of low-density lipoprotein (LDL) cholesterol. Interestingly, miR-126 was increased when LDL cholesterol was high in patients who did not have evident CAD on coronary angiography even though they have risk factors for CAD. In smokers serum cotinine levels were inversely correlated with endothelial function expressed as RH-PAT index and positively correlated with levels of metabolic parameters such as non-high-density lipoprotein (HDL) cholesterol and insulin resistance. More than half of the smokers could not completely attain smoking cessation and, thus, the RH-PAT index was not improved 8 weeks after the instruction of smoking cessation. However, changes in the RH-PAT index showed a significant correlation with those in systolic blood pressure. In smokers who completely attained smoking cessation, both RH-PAT index and plasma miR-126 values were increased. Thus, among patients with suspected CAD or subjects with coronary risk factors plasma levels of endothelium-enriched circulating miR-126 could be substantially altered. The results suggest a potential usefulness of miR-126 as a sensitive biomarker in assessing endothelial damage. Measurement of microRNA may serve as a useful tool for laboratory assays to determine high-risk patients for atherothromobotic vascular diseases.

## Background

Coronary artery disease (CAD) is both a leading cause of death and an important health threat in Asian countries as well as in European countries. Markers for CAD in circulation have been sought and identified. However, only a few markers have important clinical implications. Disease biomarkers that can appropriately evaluate the risks for CAD and the early processes of atherosclerosis are desirable. Circulating microRNAs (miRNAs) could be useful candidates as disease biomarkers including cancer. MiRNAs are a class of 20-25 nt single-stranded noncoding RNAs. MiRNAs regulate cellular functions through modulating mRNA degradation and repress translation of mRNAs. MiRNAs regulate the gene expression regarding cell growth, proliferation and apoptosis. MiRNAs are found not only in tissues, but also in body fluids including plasma. Circulating miRNAs remain in stable forms and they are resistant to endogenous RNase. Therefore, the signatures of miRNA in circulation can be useful in diagnosis, therapeutic efficacy and prognosis.

## Review

### Use of microRNA for biomedical research in thrombosis

Use of microRNA for biomedical research in thrombosis and hemostasis has provided substantial useful information. Matsui et al. evaluated a modified lentiviral delivery strategy [1]. By incorporating miRNA target sequences they succeeded in facilitated transgene expression in liver and prevented off-target expression in hematopoietic cells. This system effectively restricted transgene expression of coagulation factor VIII (FVIII) to the liver. Level of FVIII in plasma was increased to 9 % of normal level in the hemophilia A mice. Thrombin is thrombogenic and mediates inflammation. Thrombin induces activation and dysfunction of endothelial cells through NF-kB and contributes to formation of arterial thrombus. MiR-181b possesses anti-inflammatory actions and inhibits downstream NF-kB signaling in response to TNF-α. MiR-181b also inhibits upstream NF-kB signaling induced by thrombin [2].

### Dyslipidemia and microRNA

We measured circulating miRNA in CAD patients. Significant CAD was confirmed by angiography. MiRNA was also measured in patients without CAD [3]. This study was approved by our institutional ethical committee. Informed consent was obtained from all participants. Dysfunction of endothelium is implicated in early processes of atherosclerosis and thrombosis. Therefore, vascular endothelium-enriched miRNA (miR-126) that can potentially regulate angiogenesis was selected and measured. CAD patients who showed higher levels of myocardial stress marker, brain natriuretic peptide. Their average New York Heart Association class, an index of heart failure, was also higher. The prevalence of hypertension and diabetes were similar between the 2 groups. Patients without CAD showed lower prevalence of dyslipidemia. The lipid profiles were not significantly different between the 2 groups due to anti-hyperlipidemic drugs. Use of aspirin, α- blockers, angiotensin converting enzyme inhibition/angiotensin receptor blockers, statin, long-acting nitrates, insulin, and sulfonyl urea were less frequent in patients without significant CAD. Use of calcium channel blocker was not significantly different between the 2 groups. The levels of miRNA in circulation are relatively low. Thus, approach using microarray may not be sensitive enough to identify miRNAs that can serve as disease biomarkers. Therefore, quantitative RT polymerase chain reaction (qRT-PCR) analysis was utilized on RNA isolated from plasma. There are several plasma miRNAs that were previously reported to have altered levels in type 2 diabetes. We evaluated the candidate miRNAs. The analysis revealed that plasma miR-126 level of patients with CAD was comparable to the level of patients without CAD. This result indicated that miR-126 was not a specific marker for CAD. We noticed that there had been no report that level of miR-126 was influenced by low-density lipoprotein (LDL) cholesterol. The level of miR-126 was significantly decreased in CAD patients who also showed high LDL cholesterol level. The miR-126 level was increased in patients who had risk factors for CAD and high level of LDL cholesterol but did not have CAD. Taken together, our results suggested an association between dysregulated plasma miR-126 and elevated levels of LDL cholesterol in patients who have CAD.

### Smoking, endothelial function and microRNA

We also determined microRNA in middle-age male smokers [4]. Serum cotinine levels, a metabolite marker of nicotine, were inversely correlated with endothelial function expressed as reactive hyperemia peripheral arterial tonometry (RH-PAT) index and high-density lipoprotein (HDL)-cholesterol level, and positively correlated with levels of non-HDL-cholesterol and homeostasis model-insulin resistance (HOMA-IR). The smokers were encouraged to stop smoking. The RH-PAT index was not changed after 8 weeks in all subjects, because only 13 subjects could attain smoking cessation, and PH-PAT did not improve in 17 subjects who could not attain smoking cessation. However, relative percent change of systolic blood pressure was significantly correlated with relative percent change of RH-PAT index. Furthermore, subjects with decreased systolic blood pressure exhibited significantly improved the RH-PAT index. Next, smokers who completely attained smoking cessation were analyzed separately. They indicated increased RH-PAT ratio and plasma miR-126 after 8 weeks of smoking cessation. However, RH-PAT index and expression levels of miR-126 did not show significant difference between subjects who completely attained 8 weeks smoking cessation and subjects who could not attain smoking cessation. These results suggested an association between dysregulated plasma miR-126 level and vascular endothelial damage in middle-age male smokers and favorable effect of smoking cessation on recovery of endothelial function and increase in miR-126 levels.

### Platelets and microRNA for development of anti-thrombotic drugs

Bray et al. investigated the platelet transcriptome in black and white healthy subjects [5]. Platelet aggregation mediated through PAR4 thrombin receptor was greater in black subjects. Platelets from black subjects expressed lower levels of miR-376c. Lower miR-376c contributed to the higher expression of phosphatidylcholine transfer protein involved in platelet activation through thrombin. These results may imply that a genomic contribution to platelet function may differ by race. This difference may partly explain the lower survival of blacks as compared to whites after myocardial infarction due to coronary atherothrombosis. In the development of agents against thrombosis, one has to consider the effects of race.

## Conclusions

MiRNAs emerged as new biomarkers useful for diagnosis and evaluation of pathophysiology of several atherothrombotic diseases. Standardization in samples, sample processing and storage need care in the laboratory. Technical protocols may become essential for the accurate assessment of miRNAs as biomarkers.

## Abbreviations

CAD: Coronary artery disease
FVIII: Coagulation factor VIII
HDL: High-density-lipoprotein
HOMA-IR: Homeostasis model-insulin resistance
LDL: Low-density-lipoprotein
miRNA: MicroRNA
RH-PAT: Reactive hyperemia peripheral arterial tonometry
